# Association between body weight perception and intuitive eating among undergraduate students in China: the mediating role of body image

**DOI:** 10.3389/fnut.2023.1288257

**Published:** 2024-01-10

**Authors:** Yuanyuan Zhu, Jiage Gao, Qinyi Gao, Dandan Chen, Zhi Zeng

**Affiliations:** ^1^School of Nursing, Nanjing University of Chinese Medicine, Nanjing, China; ^2^Nursing Department, Nanjing Jiangning Hospital, Nanjing, China; ^3^School of Health Economics and Management, Nanjing University of Chinese Medicine, Nanjing, China

**Keywords:** intuitive eating, body weight perception, body image, undergraduate students, China

## Abstract

**Background:**

The association between body weight perception and intuitive eating among undergraduate students in China remains insufficiently understood. In the present study, we were aimed to examine the correlation between body weight perception, body image, and intuitive eating and determine whether the link between body weight perception and intuitive eating was influenced by body image.

**Methods:**

A total of 1,050 undergraduate students completed the survey. Participants provided self-reported demographic details and completed two structured scales. The Body Esteem Scale for Adolescents and Adults (BESAA) and the Intuitive Eating Scale-2 (IES-2) were employed to assess body image and intuitive eating. Analysis of the mediation model was conducted using version 4.1 of the PROCESS Macro. Results with a value of p less than 0.05 were deemed statistically significant.

**Results:**

The average age of the participants was 20.08 years (SD = 1.64). Among the students, 837 (79.7%) were female, and 212 (20.3%) were male. Body image (*r* = −0.429, *p* < 0.001) and intuitive eating (*r* = −0.313, *p* < 0.001) exhibited significant negative associations with body weight perception. Furthermore, body image showed a significant positive correlation with intuitive eating (*r* = 0.318, p < 0.001). Significant mediating effects of body image were identified concerning intuitive eating and body weight perception in the right weight (95% bootstrap CI = 0.007, 0.040) and overweight groups (95% bootstrap CI = −0.048, −0.009). The indirect effects of body image constituted 12.19% and 15.33% of the total effects of intuitive eating in these two groups.

**Conclusion:**

Although the indirect effects were not substantial, these outcomes shed light on the partial understanding of how body weight perception impacted intuitive eating via body image. Importantly, our findings emphasized the significance of body image and body weight perception, offering a novel insight for prospective interventions targeting undergraduate students.

## 1 Introduction

Dieting is the mainstream method to control or lose weight. Although dieting has advantages in weight reduction in the short term, its associations with eating disorders and psychological problems should not be ignored in the long term. Restrictions on eating frequency, amount, and variety can raise the risk of anxiety, depression, metabolic diseases (e.g., diabetes, hypertension), and even mortality (1). Consequently, there is a burgeoning interest in investigating adaptive eating behaviors like intuitive eating to mitigate these potential adverse consequences. Intuitive eating denotes an adaptive eating approach strongly tied to internal physiological signals such as hunger and satiety, rather than external cues (2). Tylka previously outlined three defining characteristics of intuitive eating. “Unconditional Permission to Eat” entails individuals not resisting or feeling no guilt about hunger, eating when hungry without labeling food as good or bad. “Eating for Physical Rather than Emotional Reasons” signifies consuming food due to physical hunger instead of negative emotions. “Reliance on Hunger and Satiety Cues” denotes individuals’ confidence in their sense of hunger and fullness, guiding their eating patterns. “Body-Food Choice Congruence” was subsequently identified as the fourth feature of intuitive eating, encompassing the selection of foods that fulfill both physical needs and psychological satisfaction, thereby promoting good health and functionality (3). Furthermore, a meta-analysis has demonstrated a negative correlation between intuitive eating and eating disorders, alongside positive associations with body image, self-esteem, and wellbeing (4). Therefore, paying attention to the status of intuitive eating can be an optional approach to promote healthy eating and mental health.

When considering intuitive eating, it becomes easier to establish a connection with body weight. Recently, several studies have detected the relationship between body weight and intuitive eating. For example, eating more intuitively is correlated with better weight status in young adults and the elderly (5–7). These findings underscore the substantial influence of body weight on dietary behavior. Simultaneously, body weight perception plays a crucial role regarding body weight. Investigations have demonstrated that body weight perception significantly impacts individuals’ lifestyles, including their exercise and dietary behaviors (8, 9). Body mass index (BMI) serves as a widely used objective measure of body weight, reflecting actual weight status. Nevertheless, body weight perception contrasts as a subjective evaluation. It encompasses an individual’s awareness of their body weight, shape, and weight status (10). Generally, individuals perceive themselves as underweight, having the right weight, or being overweight (9). While some accurately assess their weight, others experience discrepancies between body weight perception and actual body weight, with overestimation and underestimation frequently occurring (11). Those who self-perceive as underweight or overweight, or exhibit misperceptions about their body weight, seem more prone to adopting unhealthy eating behaviors and face an elevated risk of developing eating disorders (8, 12, 13). An American study showed that body weight perception surpasses BMI as a predictor for managing body weight and dietary habits (14). However, existing research fails to distinctly elucidate the relationship between body weight perception and intuitive eating. Moreover, given population disparities, undergraduate students represent a pivotal cohort in shaping appropriate body weight perception and fostering healthy eating habits (15). Hence, there exists a necessity to investigate the association between body weight perception and intuitive eating among undergraduate students. Culture should not be neglected in forming body weight perception. Numerous studies have highlighted differences in body weight perception among individuals from diverse countries (16, 17). Therefore, the present study undertook a survey in China to explore the body weight perception of Chinese undergraduate students.

The role of body image emerges as a critical factor in the investigation of intuitive eating. Body image encompasses individuals’ assessments and attitudes towards their body or appearance, including elements of perception, attitude, cognition, and behavior (18). A previous correlation analysis with Spanish adolescents has revealed that body weight perception was correlated with body image, and misperception of being overweight was associated with a poor body image (19). A systemic review comprising 97 studies has underscored a positive link between body appreciation and intuitive eating, providing substantial evidence for the relationship between body image and intuitive eating (4). In addition to the direct relationship, the mediating impact of weight and shape concerns on the association between BMI and intuitive eating exhibited statistical significance, particularly among older women. It suggests that body image serves as a mediator in the relationship between BMI and intuitive eating (7). Given the distinction between BMI and body weight perception, there is still a significant gap in research concerning the validity of the mediating model whether body weight perception influences intuitive eating via body image. Consequently, to delve into the mechanisms of intuitive eating, we investigated the connections among body weight perception, body image, and intuitive eating, and explored whether the relationship of body weight perception with intuitive eating was mediated by body image. We formulated two hypotheses designed to elucidate the pivotal processes underlying the advancement of intuitive eating among undergraduate students: (a) body weight perception exhibits a negative correlation with intuitive eating; and (b) body image mediates the associations between body weight perception and intuitive eating among undergraduate students.

## 2 Materials and methods

### 2.1 Participants and procedures

All participants were undergraduate students from the Nanjing University of Chinese Medicine. This survey was carried out utilizing a convenience sampling method from September 4, 2021, to May 23, 2022. An anonymous electronic questionnaire was edited by Questionnaire Star. The study was introduced to participants, after they signed the informed consent, they filled in the questionnaire. A total of 1,159 students voluntarily participated in the survey, with 1,050 qualified questionnaires included for statistical analysis after quality checks. Notably, 109 incomplete questionnaires were excluded from the final analysis. The authors affirm that all procedures contributing to this study complied with the Helsinki Declaration and ethical standards of relevant national and institutional committees on human experimentation. The study was approved by the Ethics Committee of Nanjing Jiangning Hospital.

### 2.2 Measurements

The questionnaire comprised two parts. The initial segment gathered self-reported demographic data from participants, including gender, age, BMI (calculated as weight in kilograms divided by the square of height in centimeters), major, living area, body weight perception, and family income. The second part incorporated two structured scales: the Body Esteem Scale for Adolescents and Adults (BESAA) alongside the Intuitive Eating Scale-2 (IES-2).

Developed by Mendelson et al. in 2001, the BESAA aims to assess body image (20) and consists of 23 items across three subscales (private general feelings about appearance, weight satisfaction, and evaluations attributed to others about one’s body and appearance), utilizing a 5-point Likert scale. Items were scored from 0 (never agree) to 4 (always agree), summing up to a total score ranging from 0 to 92. Notably, nine negative items were reverse-scored. The total score was computed by adding up the scores of each item. A higher total score indicates a more positive body image. The Cronbach’s coefficient alpha for the total scale was calculated at 0.94 (21).

Tylka and Kroon developed the original IES in 2006 and revised it in 2013 to form the IES-2 (22). This 23-item scale aims to gauge an individual’s intuitive eating tendencies. Comprising four subscales rated on a 5-point Likert scale (1 = strongly disagree, 2 = disagree, 3 = neutral, 4 = agree, 5 = strongly agree), the total score was computed by summing the items and dividing by the number of items. The Chinese version of the IES-2 yielded a Cronbach’s coefficient alpha of 0.933, with a test–retest correlation of 0.831 among undergraduate students (23).

### 2.3 Data analysis

The statistical analyzes were conducted using the Statistical Package for Social Sciences (SPSS) version 22 (SPSS, Inc., Chicago, IL, United States). Notably, no missing data was reported within the electronic questionnaire. Numerical variables were expressed as means ± standard deviations, while categorical variables were presented in quantities (proportions). The *χ*^2^- and t-tests were used to compare BESAA and IES-2 scores in different populations. Correlations were explored among the three key variables. For mediation analysis, Model 4 in version 4.1 of the PROCESS Macro plug-in authored by Hayes (24) was utilized. In this analysis, body weight perception acted as the independent variable, classified, with underweight set as the reference (25). Body image served as the mediator, intuitive eating as the dependent variable, and age, gender, and BMI functioned as covariates. To ascertain the significance of the mediation effect, a 95% bootstrap confidence interval (CI) based on 5,000 bootstrapped samples was applied. The mediation effect was significant if zero was not included in the 95% CI. A *p*-value <0.05 was considered statistically significant.

## 3 Results

### 3.1 Demographic characteristics

A total of 1,050 undergraduate students completed the cross-sectional study. The average age of the participants was 20.08 years (SD = 1.64). Notably, the majority of the participants were female (79.7%). Furthermore, 497 students (47.3%) were pursuing a medical specialty, 408 (38.9%) resided in cities, and 490 (46.8%) reported monthly family incomes exceeding 8,000 yuan. The mean BMI stood at 20.87 kg/m^2^ (SD = 3.44). 697 (66.4%) participants reported normal BMI, while only 479 (45.6%) participants considered their body weight were right. Overweight and obese students accounted for 12.4%, but 41.1% of students thought they were overweight (Table 1).

**Table 1:** Demographic characteristics and univariate analysis of body image, and intuitive eating of participants with different characteristics (*N* = 1,050).

Characteristic	*N* (%)	BESAA total score Mean ± SD	t/F	*p*	IES-2 total score Mean ± SD	t/F	*p*
Age (year)							
<20	433 (41.2)	49.95 ± 13.09	−0.188	0.851	3.33 ± 0.42	−2.206	0.028
≥20	617 (58.8)	50.11 ± 13.16			3.39 ± 0.45		
BMI (kg/m^2^)							
<18.5	223 (21.2)	54.64 ± 11.26	23.511	<0.001	3.44 ± 0.41	8.886	<0.001
18.5 ~ <24	697 (66.4)	50.30 ± 12.44			3.37 ± 0.46		
24 ~ <28	100 (9.5)	42.05 ± 12.80			3.19 ± 0.33		
≥28	30 (2.9)	36.70 ± 20.32			3.31 ± 0.47		
Gender							
Female	837 (79.7)	49.94 ± 12.89	0.495	0.621	3.35 ± 0.44	1.975	0.049
Male	212 (20.3)	50.44 ± 14.01			3.42 ± 0.43		
Major							
Medical specialty	497 (47.3)	49.40 ± 13.65	−0.797	0.426	3.35 ± 0.45	−1.336	0.182
Non-medical specialty	553 (52.7)	50.31 ± 12.63			3.38 ± 0.44		
Living area							
City	408 (38.9)	50.07 ± 13.23	1.200	0.302	3.38 ± 0.41	1.476	0.229
Town	337 (32.1)	50.80 ± 13.09			3.38 ± 0.46		
Rural area	305 (29.0)	49.19 ± 13.00			3.33 ± 0.46		
Family income/monthly (yuan)							
<2,000	44 (4.2)	46.41 ± 13.53	2.120	0.096	3.33 ± 0.48	0.884	0.449
2,000 ~ 4,999	225 (21.4)	48.91 ± 13.24			3.33 ± 0.41		
5,000 ~ 7,999	291 (27.7)	50.57 ± 13.35			3.39 ± 0.49		
≥8,000	490 (46.7)	50.58 ± 12.85			3.37 ± 0.42		
Body weight perception							
Right weight	479 (45.6)	56.71 ± 10.89	189.307	<0.001	3.53 ± 0.49	98.067	<0.001
Underweight	137 (13.0)	51.45 ± 10.76			3.36 ± 0.34		
Overweight	434 (41.4)	42.24 ± 11.79			3.18 ± 0.32		

BESAA, Body Esteem Scale for Adolescents and Adults; IES-2, Intuitive Eating Scale-2; BMI, body mass index.

Examination through *χ*^2^- and *t*-tests revealed that individuals with a BMI less than 18.5 kg/m^2^ or those with an accurate body weight perception presented higher scores in both BESAA and IES-2. Furthermore, older individuals (aged 20 years or more) and males exhibited a better degree of intuitive eating (Table 1).

### 3.2 Preliminary correlation analyzes

The mean scores for total body image and intuitive eating were 50.05 ± 13.12 and 3.37 ± 0.44, respectively. Body image (*r* = −0.429, *p* < 0.001) and intuitive eating (*r* = −0.313, *p* < 0.001) were significantly negatively related to body weight perception. Additionally, a significant positive correlation was observed between body image and intuitive eating (*r* = 0.318, *p* < 0.001).

### 3.3 Mediating effect

The mediating effect of body image between body weight perception and intuitive eating was analyzed after controlling the age, gender, and BMI among undergraduate students (Table 2). Comparatively, the underweight group revealed that body weight perception exhibited a significantly positive association with both intuitive eating (*β* = 0.398, *p* < 0.001) and body image (*β* = 0.455, *p* < 0.001) within the right weight group. Both body weight perception and body image were included in the mediation analysis. Body weight perception (β = 0.350, *p* < 0.001) and body image (*β* = 0.107, *p* = 0.002) had positive predictive effects on intuitive eating in the right weight group. Compared to the underweight group, body weight perception was significantly negatively related to intuitive eating (*β* = −0.395, *p* < 0.001) and body image (*β* = −0.567, *p* < 0.001) within the overweight group. Subsequently, both body weight perception (*β* = −0.335, *p* = 0.001) and body image (*β* = 0.107, *p* = 0.002) were included in the mediation model, ultimately exhibiting predictive effects on intuitive eating within the overweight group.

**Table 2:** Mediating effect of body image between body weight perception and intuitive eating (*N* = 1,050).

	B	*β*	SE	*t*	*p*
Reference: underweight					
Right weight/overweight → Body image					
Age	−0.193	−0.024	0.212	−0.910	0.363
Gender	−0.144	−0.004	0.897	−0.161	0.873
BMI	−0.404	−0.106	0.119	−3.396	0.001
Right weight	5.965	0.455	1.110	5.372	<0.001
Overweight	−7.433	−0.567	1.237	−6.008	<0.001
Right weight/overweight → Intuitive eating					
Age	0.006	0.023	0.008	0.785	0.432
Gender	−0.045	−0.041	0.033	−1.359	0.174
BMI	−0.001	−0.005	0.004	−0.137	0.891
Right weight	0.176	0.398	0.041	4.328	<0.001
Overweight	−0.174	−0.395	0.045	−3.855	<0.001
Right weight/overweight, body image → Intuitive eating					
Age	0.007	0.025	0.008	0.878	0.380
Gender	−0.044	−0.040	0.033	−1.349	0.178
BMI	0.001	0.007	0.004	0.197	0.844
Right weight	0.154	0.350	0.041	3.765	<0.001
Overweight	−0.148	−0.335	0.046	−3.223	0.001
Body image	0.004	0.107	0.001	3.189	0.002

BMI, body mass index.

Furthermore, the results of the bootstrapping method confirmed the significance of the indirect effects of body weight perception on intuitive eating through body image (95% bootstrap CI = 0.007, 0.040 and − 0.048, −0.009) within both the right weight and overweight groups. Since none of the 95% bootstrap CIs included zero between their lower and upper limits, the mediating effects were statistically significant for both groups. The direct effects of body weight perception on intuitive eating were measured at 0.154 and 0.148, with 95% CIs ranging from 0.080 to 0.225 and − 0.225 to −0.074 within the right and overweight groups, respectively. It was observed that the indirect effects of body image accounted for 12.19% and 15.33% of the total effects of intuitive eating within the two groups (Table 3).

**Table 3:** Mediating model examination by bootstrap (*N* = 1,050).

	Effect	BootSE	Boot CI lower limit	Boot CI upper limit	Mediating effect account
Right weight → Body image → Intuitive eating	0.021	0.008	0.007	0.040	12.19%
Right weight → Intuitive eating	0.154	0.037	0.080	0.225	87.81%
Overweight → Body image → Intuitive eating	−0.027	0.010	−0.048	−0.009	15.33%
Overweight → Intuitive eating	−0.148	0.039	−0.225	−0.074	84.67%

## 4 Discussion

This cross-sectional study aimed to examine the relationship between body weight perception, body image, and intuitive eating, as well as explore the indirect effect of body image on these parameters among undergraduate students in China. The current findings demonstrated that in addition to the direct effect on intuitive eating, body weight perception also affected intuitive eating via body image. These results provided practical significance for deepening the relationship between body weight conception, body image, and intuitive eating. Furthermore, they can serve as a guide for nurturing positive body image and promoting better intuitive eating among undergraduate students with varying body weight perceptions.

The mean total intuitive eating score was 3.37, indicating a moderate level, akin to levels reported in studies involving college students from both American and Chinese settings (26, 27). Although there has been limited research focusing on this aspect among undergraduate students, it can be seen that they have a neutral attitude toward intuitive eating. This study did identify a statistically significant difference in intuitive eating scores across two age groups, but the magnitude of this difference was minimal, possibly due to a small effect size. Combined with previous studies on different ages (e.g., adults and elderly), intuitive eating did not greatly change with age, suggesting that its overall trend is relatively stable (7, 28, 29). The body image of the students enrolled here was at a moderate level. Arslan et al. presented a lower body image score among 4th-grade children (30). This variance might be attributed to the age gap between the participants, as our current cohort comprised undergraduate students, thus likely exhibiting more mature and objective judgments regarding body image. It’s worth noting that a Romanian study reported higher body image scores compared to our investigation (21). Additionally, our study indicated a positive correlation between body weight perception and body image. This trend could possibly be explained by the fact that the Romanian study involved 427 medical students, with 65.6% perceiving themselves as having an accurate weight, whereas in our study, only 45.6% described their weight as suitable. Those who regarded themselves as having an appropriate weight tended to demonstrate higher scores on body image assessment.

Two-thirds of participants had normal BMI, whereas only 45.6% considered their body weight right. Overweight and obese participants accounted for 12.4%, but 41.1% of participants described themselves as overweight. Notably, there was a difference between the actual body weight and the body weight perception, and students were prone to perceive themselves as overweight, consistent with a previous study in which one-third of Chinese adolescents with normal BMI reported themselves as overweight (31). This result might be explained by the popular aesthetic trend that being thinner is better (31). Undergraduate students are in a sensitive period of shaping their aesthetic and judgment abilities and are easily influenced by social media. Even if they have a normal weight, they still think they are not thin enough and pursue a thinner body shape to cater to the popular aesthetic (9).

An important demonstration of the present study was that body weight perception and intuitive eating were correlated (*r* = −0.313, *p* < 0.001), and the heavier the body weight perceived, the lower level of intuitive eating. To our knowledge, this investigation marks the first attempt to explore the link between body weight perception and intuitive eating. Among students who viewed themselves as overweight or obese, there was a heightened inclination towards pursuing weight loss compared to those who regarded themselves as just the right weight, including instances of misperception regarding body weight (32). Those perceiving themselves as overweight tended to take various actions to achieve the desired weight loss. Food intake restriction is the most common way for students to manage their weight, contrary to the concept of intuitive eating. However, students not only limit the intake of highly processed and high-calorie foods but also reduce the intake of essential nutrients, leading to malnutrition and even eating disorders (33, 34). Therefore, cultivating the habit of students to establish a correct body weight perception has significance in reducing unhealthy eating and improving students’ level of intuitive eating.

Herein, we corroborated a significant indirect relationship via body image between body weight perception and intuitive eating in both the right and overweight groups, indicating that their association was partly mediated by body image. We also revealed that body weight perception was associated with body image, consistent with previous studies (19, 35, 36). Students who perceived themselves as having an appropriate weight exhibited greater contentment with their body image. Regardless of whether students accurately estimated their weight, as long as students subjectively considered that they were heavier, the more negative they were with their body image. A Brazilian study has found that a majority of women experienced dissatisfaction with their body image, a factor closely associated with their body weight perception (36). Despite the differences in population and aesthetic culture, the findings were consistent, providing more reliable evidence for the relationship between body weight perception and body image.

According to the acceptance model of intuitive eating, it is not necessarily the body weight that predicts body appreciation but the views of body acceptance by others and society (37). People are both independent individuals and members of society, and the views of others influence their perceptions and judgments. This underscores the critical importance of developing the right body weight perception. Those with higher body image scores tended to exhibit a more favorable inclination towards intuitive eating practices (7, 28). Lee et al. proposed that intuitive eating contributes to diminishing negative body image among women (38). Body image is a psychological construction. People who are satisfied with their body image will use an adaptive approach to reply to the physical cues and respect physical signals, such as hunger and satiety, rather than ignoring or restraining these internal feelings (39). Consequently, body image is an intrinsic factor that influences intuitive eating. Given the psychological and physiological benefits of intuitive eating for individuals, more research focusing on improving intuitive eating are required. Most interventions have followed 10 intuitive eating principles, including recognizing hunger and satiety cues, and awareness of emotional and stress eating (40). However, previous interventions have not included teaching participants how to treat their body weight and establish positive body image. Considering the direct and indirect impacts of body weight perception and body image on intuitive eating, integrating both variables into interventions may represent a practical and effective strategy.

However, our current study also has some limitations. Firstly, due to the utilization of a cross-sectional design, the establishment of causal relationships among body weight perception, body image, and intuitive eating was not feasible. In future, longitudinal studies will be imperative to scrutinize these causal connections in the future. Secondly, the data were obtained from self-reported measures, which may lead to response bias. Finally, although the indirect effect of body image on the relationship between body weight perception and intuitive eating was verified, its proportion was not large, suggesting that additional mediators should be explored in future studies.

Despite the above limitations, the present study also has several strengths. To our knowledge, this study was the first to examine the relationships between body weight perception, body image, and intuitive eating, providing novel and significant results to expand the existing recognition of intuitive eating among undergraduate students in China. Furthermore, our results may significantly contribute to an enhanced understanding of the underlying mechanisms shaping body weight perception and intuitive eating.

## 5 Conclusion

In summary, we demonstrated that body weight perception and body image influence intuitive eating and that body image mediate body weight perception and intuitive eating. The findings indicated that individuals perceiving themselves as overweight and expressing dissatisfaction with their body image were inclined to exhibit reduced participation in intuitive eating behaviors. These findings partially explain how body weight perception influenced intuitive eating through its association with body image. Significantly, these results highlighted the role of body image and weight perception, presenting a novel insight for prospective interventions. Interventions aimed at promoting intuitive eating among undergraduate students ought to concentrate on core aspects while integrating pertinent factors to facilitate the development of accurate body weight perceptions and foster positive body images, ultimately enhancing intuitive eating practices.

## Data availability statement

The raw data supporting the conclusions of this article will be made available by the authors, without undue reservation.

## Ethics statement

The studies involving humans were approved by Ethics Committee of Nanjing Jiangning Hospital. The studies were conducted in accordance with the local legislation and institutional requirements. The participants provided their written informed consent to participate in this study.

## Author contributions

YZ: Conceptualization, Data curation, Formal analysis, Funding acquisition, Methodology, Software, Writing – original draft, Writing – review & editing. JG: Data curation, Funding acquisition, Writing – original draft, Writing – review & editing. QG: Conceptualization, Supervision, Writing – original draft, Writing – review & editing. DC: Conceptualization, Supervision, Writing – original draft, Writing – review & editing. ZZ: Conceptualization, Supervision, Writing – original draft, Writing – review & editing.
